# A Coherence Improvement Method Based on Sub-Aperture InSAR for Human Activity Detection

**DOI:** 10.3390/s21041424

**Published:** 2021-02-18

**Authors:** Zhongbin Wang, Bingnan Wang, Maosheng Xiang, Xiaoning Hu, Chong Song, Shuai Wang, Yachao Wang

**Affiliations:** 1 National Key Laboratory of Microwave Imaging Technology, Aerospace Information Research Institute, Chinese Academy of Sciences, Beijing 100094, China; wangzhongbin17@mails.ucas.ac.cn (Z.W.); xms@mail.ie.ac.cn (M.X.); huxiaoning16@mails.ucas.ac.cn (X.H.); songchong18@mails.ucas.edu.cn (C.S.); wangshuai161@mails.ucas.edu.cn (S.W.); wangyc@aircas.ac.cn (Y.W.); 2 School of Electronic, Electrical and Communication Engineering, University of Chinese Academy of Sciences, Beijing 100094, China

**Keywords:** interferometric synthetic aperture radar (InSAR), sub-aperture InSAR, human activity detection, coherence, security monitoring

## Abstract

Human activity detection plays an important role in social security monitoring. Since human activity is very weak, it is necessary to employ the repeat-pass Interferometric Synthetic Aperture Radar (InSAR) technique to detect the potential activity between two data acquisitions; a high level of coherence is required for detection. With the object of detecting human activity of interest, this paper presents a coherence improvement approach based on sub-aperture InSAR for human activity detection. Different sub-apertures contain different scattering information of the target, as they represent the backscatter of the target from a different range of angles. Integrating corresponding sub-aperture interferometric results can improve the coherence between two complex images compared to the entire synthetic aperture, as well as removing a little disturbance in some circumstances. To validate the method presented in this paper, the actual airborne Ka-band frequency modulated continuous wave (FMCW) InSAR data acquired by the Aerospace Information Research Institute, Chinese Academy of Sciences (AIRCAS) are utilized. The experimental results demonstrate that the proposed method can effectively improve the coherence between two complex SAR images and can validly detect human activity of interest.

## 1. Introduction

SAR is a coherent imaging system that can work all day and in all kinds of weather. It can acquire the temporal images of an area conveniently. The most basic use of SAR is image formation. As the resolution of the SAR system improves, its applications in other areas are further developing [1,2,3]. Particularly, SAR contributes to target recognition. With the development of society, security defense, ground search and surveillance [4], threat activity detection on the ground [5], ground activity management and other human activities are becoming important topics of social security [6]. These human activities on the ground can be identified with SAR. Besides, in military applications, the need to manage military sites and detect artificial targets in various climatic environments is also growing [7].

The traditional SAR system has high requirements in terms of the load capacity and flight stability of the platform since it is large in size, heavy in weight and is characterized by complex system implementation. Therefore, its application scope has been limited by the huge operating and maintenance costs. The frequency modulated continuous wave (FMCW) has the characteristics of a large bandwidth, light weight, small size and low cost. With the development of numerous small platforms such as unmanned aerial vehicles and aircraft, FMCW SAR and its applications have attracted the attention of many researchers around the world [8,9,10]. This paper discusses the application of FMCW SAR in the detection of human activity, and a coherence improvement method based on sub-aperture InSAR is discussed for human activity detection as well.

Human activity detection makes use of repeat-pass Interferometric Synthetic Aperture Radar (InSAR) technology to detect, locate and extract potential activities between two observations [11,12,13], such as vehicle tracks, footprints, human grazing and other activities. These subtle activities cannot be observed in the amplitude image directly, so they need to be captured with information that is more sensitive. SAR system is distinct from other sensors in that it can obtain not only the amplitude information of the target, but also the phase information. There are normally two detection methods [14]: one is the incoherent change detection method based on amplitude information—this method can only detect large-scale activities, such as parking cars, truck track and so on, but it cannot detect small-scale activities due to the limitation of resolution; the other is the coherent change detection (CCD) method based on phase information, which can detect small-scale activities [15,16,17] such as human footprints, car track, grazing and other very subtle human activities. This is explained by the fact that the phase information acquired by the SAR system is very sensitive to subtle changes. Generally, the change of half of the wavelength level is large enough to cause complete decorrelation [18]. Considering the influence of noise and other factors, CCD can at least detect changes in the wavelength scale, which means that the CCD method plays an important role in detecting human activities and other small changes.

However, the repeat-pass InSAR has problems such as its temporal baseline, non-parallel flight route and real-time varying attitude and position [19,20], which makes the coherence of repeat-pass interferometry image pairs very low. Besides, light and small carrier platforms installed with FMCW SAR are more sensitive to atmospheric disturbances. The stability of flight is poor, resulting in serious errors in trajectory and attitude, which make it difficult to obtain interferometry image pairs directly to detect human activities. In addition, human activities are very subtle. Both the size and range of the activities are very small and barely result in a change in amplitude information. In practice, since the high-frequency SAR system has a concise description regarding scene changes, it is usually the first choice to detect human activity clearly and reliably. Meanwhile, a high frequency also means high resolution; this results in the traditional processing approach no longer being suitable for human activity detection and brings challenges for the processing of human activity detection as well.

Sub-aperture processing has many applications in different contexts. Many researchers have conducted studies into SAR sub-aperture processing. N. Veneziani and D. Derauw utilized sub-aperture processing, achieving repeat raw data focusing [21,22]. F. Bovenga and N. Veneziani utilized sub-aperture processing for coherent target detection [23,24]. In addition, sub-aperture processing makes a difference in SAR image autofocusing [25,26], motion compensation [27], the speckle filtering of SAR images [28], highly squinted SAR imaging [29], etc. In contrast to the above applications, this paper utilizes sub-aperture InSAR processing for human activity detection.

Utilizing SAR to detect human activity requires careful data collection, accurate SAR image processing and correct estimation. Based on these principles, this paper combines sub-aperture processing with traditional coherent processing to improve the coherence between repeat-pass interferometry image pairs, which can increase the reliability of the detection of human activities. The results of different sub-apertures represent the backscattering information of the target in a different range of azimuth angles [30], and the sub-apertures determine which pulses participate in creating the corresponding pixel. The corresponding sub-apertures of the repeat-pass interferometry image pairs are used for interferometric processing to obtain the coherence of different ranges of angles. Interferometric results of each sub-aperture can be integrated to improve the coherence between the repeat-pass interferometry image pairs, which is very beneficial for the detection of human activity and other related applications. At the same time, sub-aperture processing can eliminate the influence of speckle to a certain extent and reduce the influence of shadow areas on coherence. In total, sub-aperture InSAR processing can improve the coherence of the scene, improving human activity detection.

In this paper, we present a coherence improvement method based on sub-aperture InSAR for human activity detection. In Section 2 of this paper, we present the sub-aperture division and the derivation of the coherence improvement method. Section 3 introduces the experiment and the implementation of the method. Section 4 presents the experimental results of the proposed method. In Section 5, this paper is concluded, and a discussion of this work is provided.

## 2. Methods

The real-time varying flight trajectory and attitude of repeat-pass InSAR observation geometry imposes challenges to obtaining high-quality detection results. This section introduces a coherence improvement method based on sub-aperture InSAR. Here, sub-aperture division and the coherence improvement method are presented.

### 2.1. Sub-Aperture Division

The geometry of repeat-pass observation collects the scattering information of the target in different moments. This information is crucial to analyze the state of the target and includes factors such as the terrain of the scene, deformation and the change of the scene. Human activity is related to a change between two observations. Since human activity is very subtle and small in size, higher coherence is required to detect it more reliably.

As the raw echo of SAR received in each pulse repetition period is the superposition of the echo signals of all target points in the range of entire beam radiation, it is impossible to distinguish a single target point in the time domain, which results in large residual motion error for the entire aperture. The raw echo records the Doppler history of the target point in the entire synthetic aperture time, and the range of observation angles in the azimuth corresponds to the Doppler frequency. The movement of the observation platform causes the azimuth beam to be reduced to a small angle [31]. If the entire beam is used for processing, this will result in a larger error. At this point, the entire beam needs to be divided into multiple sub-apertures for processing, so that only pulses within a specific range of angles can be used to create corresponding pixels, rather than the entire beam range.

**Figure 1:**
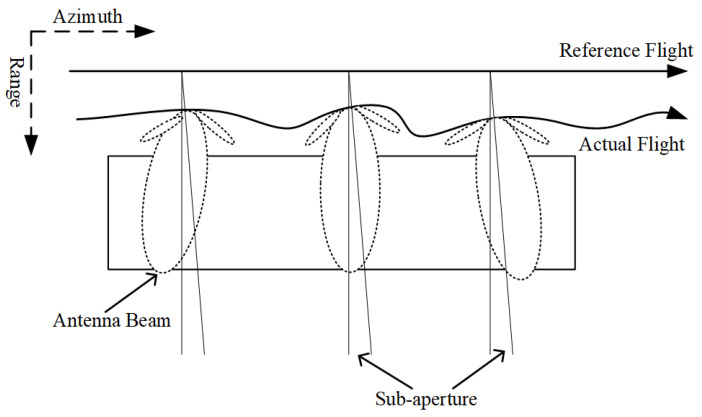
Antenna beam squinted during flight.

The sub-aperture image only obtains the scattering information within a specific angle range of the beam, and any pulses outside the angle of sub-aperture are not used for imaging, meaning that the corresponding synthetic aperture time is short, and only the error at the center frequency of the sub-aperture is used for compensation, which can make the residual error significantly smaller. For the two images of repeat-pass observations, the squinting angles of the azimuth beam caused by the movement of the platform are quite different. Intuitively, the direction of the antenna when pointing in the azimuth direction is different, as shown in Figure 1, which leads to a different range of Doppler frequency. For example, for the Ka-band airborne system in which the carrier frequency is 34.6 GHz and velocity is 31.6 m/s, the antenna’s pointing bias of 0.5${}^{\circ }$ in the azimuth direction causes a 63.6 Hz difference in Doppler frequency. In order to decrease the influence of Doppler decorrelation on repeat-pass interferometry image pairs, it is crucial to perform Doppler filtering and sub-aperture processing [32]. In general, according to [33], the entire aperture is equally divided into four sub-apertures.

**Figure 2:**
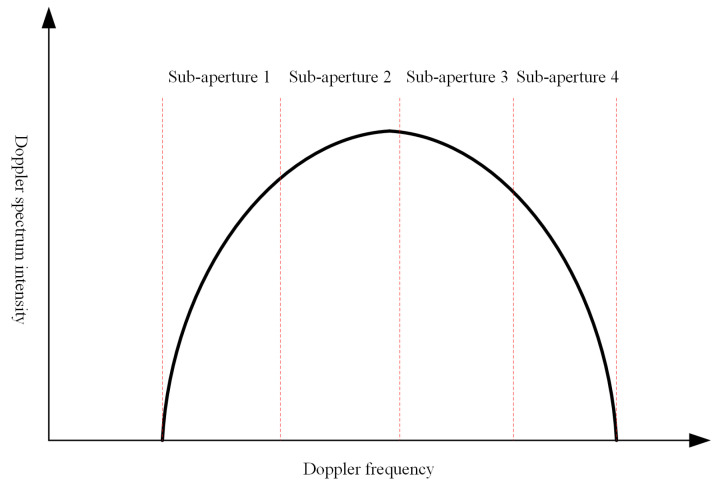
The diagram of sub-aperture division.

Sub-aperture processing defines the imaging beam width; that is, the range of frequency in the Doppler domain. This problem is further illustrated in Figure 2. The acquired results of the corresponding sub-aperture can be utilized for interferometric processing. For repeat-pass InSAR processing, sub-aperture processing can reduce the coherence loss, which is more conducive to the detection of subtle human activity.

### 2.2. Coherence Improvement Method

Human activity involves very small-scale changes, and reliable detection requires high-quality data sources and high-level processing technology. The method based on amplitude information is suitable for detecting large-scale human activity, but it is usually difficult to accurately detect subtle human activity. However, the phase information is very sensitive to changes [34], which is suitable for detecting subtle human activity. Therefore, the detection method based on phase information, called the coherent change detection method, is usually adopted.

Assuming that the two single-look complex SAR images acquired by the repeat-pass observation are $X_{1k}$ and $X_{2k}$, after coherent processing, the coherence between the two images is defined as the magnitude of the normalized complex cross-correlation coefficient. This is given as follows [35]:

(1) $$\gamma =\left|\frac{E\left[X_{1k}^{H}X_{2k}\right]}{\sqrt{E\left[{X_{1k}}^{2}\right]E\left[{X_{2k}}^{2}\right]}}\right|\;$$

where $E\left[\cdot \right]$ is the expectation operator and $A^{H}$ denotes the conjugate of variable A. In practice, the coherence in Equation (1) is computed by a spatial averaging on N samples as [36,37]

(2) $$\gamma =\left[\frac{\Sigma_{N}X_{1k}^{H}X_{2k}}{\sqrt{\Sigma_{N}{X_{1k}}^{2}\Sigma_{N}{X_{2k}}^{2}}}\right]\;$$

**Figure 3:**
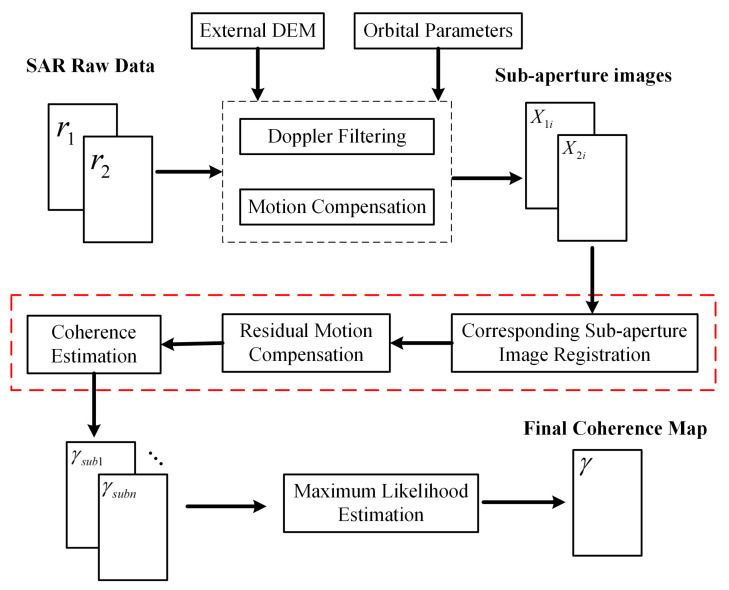
The whole process of coherence improvement method based on sub-aperture Interferometric Synthetic Aperture Radar (InSAR).

The whole process of the method based on sub-aperture InSAR is shown in Figure 3. The processing mainly includes five steps. First, using SAR raw data, an external Digital Elevation Map (DEM) and orbital parameters, motion compensation and Doppler filtering operations are performed to obtain sub-aperture images. Next, using sub-aperture image pairs through the maximum complex correlation method, corresponding sub-aperture image registrations are obtained. Then, the residual motion error estimation and compensation are performed for the corresponding registered sub-aperture image pairs. The coherence maps are then obtained with Equation (2). Finally, the final coherence map is obtained through maximum likelihood estimation operation among multiple sub-aperture coherence maps.

The sub-aperture image obtains the scattering information of the target in a different range of azimuth angles. The collection of repeat-pass observation can be described through the vector $r=\left[r_{1},r_{2}\right]$. After interferometry image formation, the corresponding sub-aperture image pairs $\left(X_{1i},X_{2i}\right)$ are acquired, where the index i=1,…,n represents various sub-apertures for the master and slave images.

This paper starts with the simplest model, where the master and slave images are divided into two sub-apertures. We start with the following model [38]:

(3) $$\begin{array}{c} X_{1k_{sub1}}=C_{0k_{sub1}}+n_{1k_{sub1}}\\ X_{1k_{sub2}}=C_{0k_{sub2}}+n_{1k_{sub2}}\\ X_{2k_{sub1}}=\alpha_{sub1}C_{0k_{sub1}}e^{j\varphi_{sub1}}+\sqrt{1-\alpha_{sub1}^{2}}C_{1k_{sub1}}+n_{2k_{sub1}}\\ X_{2k_{sub2}}=\alpha_{sub2}C_{0k_{sub2}}e^{j\varphi_{sub2}}+\sqrt{1-\alpha_{sub2}^{2}}C_{1k_{sub2}}+n_{2k_{sub2}} \end{array}$$

where $X_{1k_{sub1}}$, $X_{1k_{sub2}}$, $X_{2k_{sub1}}$ and $X_{2k_{sub2}}$ are the *k*th values of in the two sub-apertures of master and slave images. The parameters $\left[\alpha_{sub1}\;\alpha_{sub2}\right]\in C^{2}$ are the measures of change in the two corresponding sub-apertures. This is an estimation of human activity occurring between the repeat-pass observations. The parameters $\left[C_{0k_{sub1}}\;C_{0k_{sub2}}\right]\in C^{n\times n\times 2}$ and $\left[C_{1k_{sub1}}\;C_{1k_{sub2}}\right]\in C^{n\times n\times 2}$ are the parts of images that showed no change and changes between repeat-pass observations, respectively. The terms $\left[\varphi_{sub1}\;\varphi_{sub2}\right]\in \left[-\pi ,\pi \right]$ are the interferometric phases between the repeat-pass sub-aperture image pairs. This phase terms are affected by the difference in the geometry of observation and terrain height. In addition, the term $\left[n_{1k_{sub1}}n_{1k_{sub2}}\right]$ and $\left[n_{2k_{sub1}}n_{2k_{sub2}}\right]$ are the additive thermal noise of the SAR system. The complex-valued variables of the model in Equation (3) are independent and identically distributed (i.i.d.) zero-mean Gaussian random variables [39] with the following relationships:

(4) $$\begin{array}{c} E\left({\left|C_{0k_{sub1}}\right|}^{2}\right)=E\left({\left|C_{0k_{sub2}}\right|}^{2}\right)=\sigma_{c}^{2}\\ E\left({\left|C_{1k_{sub1}}\right|}^{2}\right)=E\left({\left|C_{1k_{sub2}}\right|}^{2}\right)=\sigma_{c}^{2}\\ E\left({\left|n_{1k_{sub1}}\right|}^{2}\right)=E\left({\left|n_{1k_{sub2}}\right|}^{2}\right)=\sigma_{n_{1}}^{2}\\ E\left({\left|n_{2k_{sub1}}\right|}^{2}\right)=E\left({\left|n_{2k_{sub2}}\right|}^{2}\right)=\sigma_{n_{2}}^{2} \end{array}$$

where $E\left[\cdot \right]$ is the expectation operator. Then, combining Equations (3) and (4), we can write the following:

(5) $$\begin{array}{c} E\left({\left|X_{1k_{sub1}}\right|}^{2}\right)=E\left\{\frac{\sum_{k=1}^{N}{\left|X_{1k_{sub1}}\right|}^{2}}{N}\right\}=\sigma_{c}^{2}+\sigma_{n_{1}}^{2}\\ E\left({\left|X_{1k_{sub2}}\right|}^{2}\right)=E\left\{\frac{\sum_{k=1}^{N}{\left|X_{1k_{sub2}}\right|}^{2}}{N}\right\}=\sigma_{c}^{2}+\sigma_{n_{1}}^{2}\\ E\left({\left|X_{2k_{sub1}}\right|}^{2}\right)=E\left\{\frac{\sum_{k=1}^{N}{\left|X_{2k_{sub1}}\right|}^{2}}{N}\right\}=\sigma_{c}^{2}+\sigma_{n_{2}}^{2}\\ E\left({\left|X_{2k_{sub2}}\right|}^{2}\right)=E\left\{\frac{\sum_{k=1}^{N}{\left|X_{2k_{sub2}}\right|}^{2}}{N}\right\}=\sigma_{c}^{2}+\sigma_{n_{2}}^{2} \end{array}$$

where *N* is the number of samples in the window.

Equation (5) presents the observation information in a local area of SAR images. The parameter $\sigma_{c}^{2}$ is the scattering power of the target in the local area. Both the scattering power and the noise value can vary in different areas. Without loss of generality, we assume the noise value is constant over the entire scene [38]. The repeat-pass sub-aperture SAR images rewritten in vector form are as follows:

(6) $$X_{k_{sub1}}=\left(\begin{array}{c} X_{1k_{sub1}}\\ X_{2k_{sub1}} \end{array}\right)=C_{0k_{sub1}}\left(\begin{array}{c} 1\\ \alpha_{sub1}e^{j\varphi_{sub1}} \end{array}\right)+C_{1k_{sub1}}\left(\begin{array}{c} 0\\ \sqrt{1-\alpha_{sub1}^{2}} \end{array}\right)+\left(\begin{array}{c} n_{1k_{sub1}}\\ n_{2k_{sub1}} \end{array}\right)$$

and

(7) $$X_{k_{sub2}}=\left(\begin{array}{c} X_{1k_{sub2}}\\ X_{2k_{sub2}} \end{array}\right)=C_{0k_{sub2}}\left(\begin{array}{c} 1\\ \alpha_{sub2}e^{j\varphi_{sub2}} \end{array}\right)+C_{1k_{sub2}}\left(\begin{array}{c} 0\\ \sqrt{1-\alpha_{sub2}^{2}} \end{array}\right)+\left(\begin{array}{c} n_{1k_{sub1}}\\ n_{2k_{sub1}} \end{array}\right)$$

where the covariances of corresponding sub-aperture images are as follows:

(8) $$Q_{sub1}=E\left(X_{k_{sub1}}X_{k_{sub1}}^{H}\right)=\left(\begin{array}{c} \sigma_{c}^{2}+\sigma_{n_{1}}^{2}\\ \alpha_{sub1}\sigma_{c}^{2}e^{j\varphi_{sub1}} \end{array}\right.\left.\begin{array}{c} \alpha_{sub1}\sigma_{c}^{2}e^{-j\varphi_{sub1}}\\ \sigma_{c}^{2}+\sigma_{n_{2}}^{2} \end{array}\right)$$

and

(9) $$Q_{sub2}=E\left(X_{k_{sub2}}X_{k_{sub2}}^{H}\right)=\left(\begin{array}{c} \sigma_{c}^{2}+\sigma_{n_{1}}^{2}\\ \alpha_{sub2}\sigma_{c}^{2}e^{j\varphi_{sub2}} \end{array}\right.\left.\begin{array}{c} \alpha_{sub2}\sigma_{c}^{2}e^{-j\varphi_{sub2}}\\ \sigma_{c}^{2}+\sigma_{n_{2}}^{2} \end{array}\right)$$

where the superscript *H* denotes the conjugate operator. The joint conditional probability density function of two sub-aperture image pairs is given by the followings:

(10) $$P_{sub1}\left(X|\varphi_{sub1},\alpha_{sub1}\right)=\frac{1}{\pi^{2N}{\left|Q_{sub1}\right|}^{N}}e^{-\sum_{k=1}^{N}X_{k_{sub1}}^{H}Q_{sub1}^{-1}X_{k_{sub1}}}$$

and

(11) $$P_{sub2}\left(X|\varphi_{sub2},\alpha_{sub2}\right)=\frac{1}{\pi^{2N}{\left|Q_{sub2}\right|}^{N}}e^{-\sum_{k=1}^{N}X_{k_{sub2}}^{H}Q_{sub2}^{-1}X_{k_{sub2}}}$$

where $Q_{sub1}^{-1}=Q_{a_{sub1}}{\left|Q_{sub1}\right|}^{-1}$ and $Q_{sub2}^{-1}=Q_{a_{sub2}}{\left|Q_{sub2}\right|}^{-1}$ in Equations (10) and (11), respectively. $Q_{a_{sub1}}$ and $Q_{a_{sub2}}$ are the adjoint matrix of $Q_{sub1}$ and $Q_{sub2}$, respectively. $\left|Q_{sub1}\right|$ and $\left|Q_{sub2}\right|$ are the determinants of $Q_{sub1}$ and $Q_{sub2}$, respectively. They are given by

(12) $$Q_{a_{sub1}}=\left(\begin{array}{c} \sigma_{c}^{2}+\sigma_{n_{1}}^{2}\\ -\alpha_{sub1}\sigma_{c}^{2}e^{j\varphi_{sub1}} \end{array}\right.\left.\begin{array}{c} -\alpha_{sub1}\sigma_{c}^{2}e^{-j\varphi_{sub1}}\\ \sigma_{c}^{2}+\sigma_{n_{2}}^{2} \end{array}\right)\;Q_{a_{sub2}}=\left(\begin{array}{c} \sigma_{c}^{2}+\sigma_{n_{1}}^{2}\\ -\alpha_{sub2}\sigma_{c}^{2}e^{j\varphi_{sub2}} \end{array}\right.\left.\begin{array}{c} -\alpha_{sub1}\sigma_{c}^{2}e^{-j\varphi_{sub2}}\\ \sigma_{c}^{2}+\sigma_{n_{2}}^{2} \end{array}\right)$$

and

(13) $$\begin{array}{c} \left|Q_{sub1}\right|=\left(\sigma_{c}^{2}+\sigma_{n_{1}}^{2}\right)\left(\sigma_{c}^{2}+\sigma_{n_{2}}^{2}\right)-\alpha_{sub1}^{2}\sigma_{c}^{4}\\ \left|Q_{sub2}\right|=\left(\sigma_{c}^{2}+\sigma_{n_{1}}^{2}\right)\left(\sigma_{c}^{2}+\sigma_{n_{2}}^{2}\right)-\alpha_{sub2}^{2}\sigma_{c}^{4} \end{array}$$

The maximum likelihood (ML) estimation of $\left(\alpha_{sub1},\alpha_{sub2}\right)$ maximizes the value of Equations (10) and (11), regarding $\left(\varphi_{sub1},\varphi_{sub2}\right)$ as unknown parameters. The log likelihoods of Equations (10) and (11) are provided below:

(14) $$\ln\left\{P_{sub1}\left(X|\varphi_{sub1},\alpha_{sub1}\right)\right\}=-2N\ln\pi -N\ln\left|Q_{sub1}\right|-\sum_{k=1}^{N}X_{k_{sub1}}^{H}Q_{sub1}^{-1}X_{k_{sub1}}$$

and

(15) $$\ln\left\{P_{sub2}\left(X|\varphi_{sub2},\alpha_{sub2}\right)\right\}=-2N\ln\pi -N\ln\left|Q_{sub2}\right|-\sum_{k=1}^{N}X_{k_{sub2}}^{H}Q_{sub2}^{-1}X_{k_{sub2}}$$

Then, the partial operator of the log likelihoods above is given as

(16) $$\begin{array}{cc} \frac{-N\frac{d\left(\left|Q_{sub1}\right|\right)}{d\alpha_{sub1}}}{\left|Q_{sub1}\right|}-\sum_{k=1}^{N}\left(X_{k_{sub1}}^{H}\left(\left(\frac{d\left(Q_{a_{sub1}}\right)}{d\alpha_{sub1}}\right){\left|Q_{sub1}\right|}^{-1}\right.\right.\; & \\ \left.\left.\;-Q_{a_{sub1}}{\left|Q_{sub1}\right|}^{-2}\left(\frac{d\left(\left|Q_{sub1}\right|\right)}{d\alpha_{sub1}}\right)\right)X_{k_{sub1}}\right)=0 & \end{array}$$

and

(17) $$\begin{array}{cc} \frac{-N\frac{d\left(\left|Q_{sub2}\right|\right)}{d\alpha_{sub2}}}{\left|Q_{sub2}\right|}-\sum_{k=1}^{N}\left(X_{k_{sub2}}^{H}\left(\left(\frac{d\left(Q_{a_{sub2}}\right)}{d\alpha_{sub2}}\right){\left|Q_{sub2}\right|}^{-1}\right.\right.\; & \\ \;\left.\left.-Q_{a_{sub2}}{\left|Q_{sub2}\right|}^{-2}\left(\frac{d\left(\left|Q_{sub2}\right|\right)}{d\alpha_{sub2}}\right)\right)X_{k_{sub2}}\right)=0 & \end{array}$$

where $\frac{d}{d\alpha }\left(\cdot \right)$ represents the partial operator with respect to α. The maximum likelihood estimation of the phase item $\left[\varphi_{sub1}\;\varphi_{sub2}\right]$ is given as follows [40]:

(18) $${\hat{\varphi }}_{ML_{sub1}}\;=∠\left\{\sum_{k=1}^{N}X_{1k_{sub1}}^{H}X_{2k_{sub1}}\right\}\;\mathrm{and}\;{\hat{\varphi }}_{ML_{sub2}}\;=∠\left\{\sum_{k=1}^{N}X_{1k_{sub2}}^{H}X_{2k_{sub2}}\right\}$$

where the symbol *∠* represents the phase of the complex data. ${\hat{\varphi }}_{ML_{sub1}}$ and ${\hat{\varphi }}_{ML_{sub2}}$ represent the maximum likelihood estimation of $\varphi_{sub1}$ and $\varphi_{sub2}$ respectively. Equations (8), (9), (12), (13) and (18) are substituted into Equations (16) and (17); after several calculations, the following estimation results of $\left(\alpha_{sub1},\alpha_{sub2}\right)$ can be derived.

(19) $${\hat{\alpha }}_{sub1}=\frac{\left|X_{1k_{sub1}}^{H}X_{2k_{sub1}}\right|}{N\sigma_{c}^{2}}\;\mathrm{and}\;{\hat{\alpha }}_{sub2}=\frac{\left|X_{1k_{sub2}}^{H}X_{2k_{sub2}}\right|}{N\sigma_{c}^{2}}$$

where ${\hat{\alpha }}_{sub1}$ and ${\hat{\alpha }}_{sub2}$ represent the estimation of $\alpha_{sub1}$ and $\alpha_{sub2}$ respectively. It can be seen from Equation (19) that the parameter $\sigma_{c}^{2}$ is required for the estimation. This parameter can be estimated from the SAR image with the target scene. According to Equation (5), the parameter $\sigma_{c}^{2}$ can be defined as

(20) $${\hat{\sigma }}_{c}^{2}=\frac{\sum_{k=1}^{N}{\left|X_{1k_{sub1}}\right|}^{2}+\sum_{k=1}^{N}{\left|X_{2k_{sub1}}\right|}^{2}-N\sigma_{n_{1}}^{2}-N\sigma_{n_{2}}^{2}}{2N}$$

where ${\hat{\sigma }}_{c}^{2}$ is a maximum likelihood estimation of $\sigma_{c}^{2}$, and the noise value of the images can be obtained from system design instruction or estimated from the images over neighboring pixels [41].

Combining Equations (19) and (20), the maximum likelihood estimation of $\left(\alpha_{sub1},\alpha_{sub2}\right)$ is given as [38]

(21) $${\hat{\alpha }}_{sub1}=\frac{2\left|X_{1k_{sub1}}^{H}X_{2k_{sub1}}\right|}{\sum_{k=1}^{N}{\left|X_{1k_{sub1}}\right|}^{2}+\sum_{k=1}^{N}{\left|X_{2k_{sub1}}\right|}^{2}-N\sigma_{n_{1}}^{2}-N\sigma_{n_{2}}^{2}}$$

and

(22) $${\hat{\alpha }}_{sub2}=\frac{2\left|X_{1k_{sub2}}^{H}X_{2k_{sub2}}\right|}{\sum_{k=1}^{N}{\left|X_{1k_{sub2}}\right|}^{2}+\sum_{k=1}^{N}{\left|X_{2k_{sub2}}\right|}^{2}-N\sigma_{n_{1}}^{2}-N\sigma_{n_{2}}^{2}}$$

Finally, the maximum likelihood estimator (MLE) based on sub-apertures for human activity detection is provided as follows:

(23) $$\hat{\alpha }\left(x,y\right)=MLE\left({\hat{\alpha }}_{sub1}\left(x,y\right),{\hat{\alpha }}_{sub2}\left(x,y\right)\right)$$

In particular, if the test scene has a high signal-to-noise ratio (SNR) and the scattering powers between repeat-pass images are $\sum_{k=1}^{N}{\left|X_{1k_{sub1}}\right|}^{2}≅\sum_{k=1}^{N}{\left|X_{2k_{sub1}}\right|}^{2}$, Equations (21) and (22) will be equal to Equation (2).

(24) $$\begin{array}{c} {\hat{\alpha }}_{sub1}\;=\;\frac{2\left|X_{1k_{sub1}}^{H}X_{2k_{sub1}}\right|}{\sum_{k=1}^{N}{\left|X_{1k_{sub1}}\right|}^{2}+\sum_{k=1}^{N}{\left|X_{2k_{sub1}}\right|}^{2}}≅\frac{\left|X_{1k_{sub1}}^{H}X_{2k_{sub1}}\right|}{\sqrt{\sum_{k=1}^{N}{\left|X_{1k_{sub1}}\right|}^{2}}\sum_{k=1}^{N}{\left|X_{2k_{sub1}}\right|}^{2}}\;=\;{\hat{\gamma }}_{sub1}\\ {\hat{\alpha }}_{sub2}\;=\;\frac{2\left|X_{1k_{sub2}}^{H}X_{2k_{sub2}}\right|}{\sum_{k=1}^{N}{\left|X_{1k_{sub2}}\right|}^{2}+\sum_{k=1}^{N}{\left|X_{2k_{sub2}}\right|}^{2}}≅\frac{\left|X_{1k_{sub2}}^{H}X_{2k_{sub2}}\right|}{\sqrt{\sum_{k=1}^{N}{\left|X_{1k_{sub2}}\right|}^{2}}\sum_{k=1}^{N}{\left|X_{2k_{sub2}}\right|}^{2}}\;=\;{\hat{\gamma }}_{sub2} \end{array}$$

where the symbol ≅ represents an approximately equal operator. In this case, the maximum likelihood estimator based on sub-apertures for human activity detection can be transformed into the following form:

(25) $$\hat{\gamma }\left(x,y\right)=MLE\left({\hat{\gamma }}_{sub1}\left(x,y\right),{\hat{\gamma }}_{sub2}\left(x,y\right)\right)$$

If the interferometric results from multiple apertures are integrated as above, higher coherence results can be obtained for a stationary scene.

The traditional processing utilizes the scattering information of the target in the entire synthetic aperture, meaning that residual motion errors will accumulate and cause a loss of coherence. The sub-aperture interferometric processing method can effectively avoid the accumulation of residual motion error and reduce the loss of coherence. In addition, a low signal-to-noise ratio area appears as decorrelation in the large aperture processing, such as a shadowed area, but it can show high coherence in some sub-apertures and low coherence in some sub-apertures after sub-aperture interferometric processing. This is because the coherence of the target changes due to the different range of angles in the azimuth direction. The coherence map obtained by the sub-aperture processing can improve the coherence of the target area.

In short, there are numerous sources of decorrelation for repeat-pass observation, especially for airborne platforms. Nevertheless, the detection of human activity requires high-quality coherence coefficient maps. Thus, it is crucially important to develop a more optimized coherence improvement method for human activity detection. Adopting the method in this paper can improve the coherence between the two SAR image pairs, which could allow the detection of potential human activities more reliably.

## 3. Experiment and Implementation

In order to verify the effectiveness of the method in this paper, we utilized actual airborne repeat-pass InSAR data for processing. The data were obtained in September 2018 by the Ka-band FMCW InSAR system of the Aerospace Information Research Institute, Chinese Academy of Sciences. The experimental scenes were rich in terms of the types of objects. Ground cooperation experiments were carried out for the detection of human activities.

### 3.1. Experiment

Human activity detection utilizes the repeat-pass InSAR technique to detect potential human activities on the ground, which requires two-pass or multi-pass data before and after human activities occur to capture this change. In order to detect human activity reliably, high-quality repeat-pass InSAR data are required; that is, the experimental data are required to have high coherence between two InSAR image pairs. This means that the coherence of the data should be maintained as much as possible in the process of data collection and data processing to reduce the loss of coherence. When acquiring data, it is necessary for the flight path to be along the same route or as close as possible to the previous route. However, it is difficult to maintain the repeated flight path as a result of the limitations of flight and navigation levels under actual flight conditions. It is necessary to keep the flight route in a tube-shaped area with a diameter of 5 m or even smaller. The distance between the centerline of the two tubes should be kept as constant as possible. In addition, it is best to make the attitude of the aircraft vary within a small range or even invariant to increase the likelihood of obtaining data with higher coherence. All of this is essential for the correct detection of human activity on the ground.

For the human activity detection experiment, the high-resolution airborne Ka-band FMCW InSAR system developed by the Aerospace Information Research Institute, Chinese Academy of Sciences was used to carry out the corresponding ground cooperation experiment. Finally, we obtained rare and valuable experimental data for the detection of human activity. The main parameters of the system are shown in Table 1.

When acquiring the experimental FMCW InSAR data, a bare ground was selected as the target experimental scene, meaning that we could detect the actual human activities more reliably. On the first day of the flight, several small grooves were made in a small area before the flight test, which was done to verify the idea of detecting it from the phase directly. Apart from this, no other changes were made to the target scene. In order to simulate some subtle human activities over the experimental scene, the experimenter arranged a large number of tracks in the test scene as activities before the flight test on the second day, such as people walking back and forth, artificially turning soil, making artificial grooves and motorcycle tracks, as well as tricycle tracks, etc. In short, a variety of ground activities were arranged in the experimental scene.

### 3.2. Processing Framework

In order to obtain reliable human activity detection results, high-quality data sources, accurate data processing and precise coherence estimation are required. Extremely complex processing is required to obtain very good results, especially with actual airborne InSAR data sources. Here, we present some important steps for human activity detection.

Human activity detection depends on the coherence between repeat-pass observation images. Thus, the difference in the collection geometry of the repeat-pass image pairs used for human activity detection should be minimized. Since it is impossible to fly the same flight path in two observations, the flight tracks need to be designed carefully in advance to ensure the quality of raw data.

Since repeat-pass InSAR observation has a separate flight track, the range-variant, azimuth-variant and topography-variant motion errors are independent. In addition, the residual motion errors are different in two InSAR images due to the different attitude and position in each pass. To compensate for these motion errors, complicated motion compensation processing needs to be performed on the InSAR image pairs. Then, the sub-aperture repeat-pass InSAR image pairs with motion compensation can be realized. Image registration is performed subsequently. In this step, the registration method based on the maximum complex coherence coefficient criterion is applied to obtain the registration results between sub-aperture InSAR image pairs.

However, some residual motion errors unavoidably persist in InSAR image pairs after image registration processing. These residual motion errors may be caused by an error of measuring equipment, error introduced by processing, different attitudes between InSAR image pairs and high-order temporal baselines. The coherence between InSAR image pairs will be reduced without compensating for this residual errors. The multisquint technique [42] is adopted to compensate for the residual motion errors.

After the above-mentioned coherent processing, the sub-aperture InSAR image pairs are obtained to perform coherence estimation. The corresponding sub-aperture InSAR coherence coefficient maps are obtained by Equation (2). Different coherence coefficient maps of sub-aperture image pairs present distinct coherent scattering information about the target. Then, the final coherence results can be obtained with Equation (25).

In the end, the final coherence results acquired above can be used to detect potential human activity. The performance of the coherence improvement method can also be validated by the results of human activity detection.

## 4. Results

The actual airborne data collected by the Ka-band FMCW InSAR system of the Aerospace Information Research Institute, Chinese Academy of Sciences were used to verify the performance of the method proposed in this paper. In order to verify the performance of the method in this paper, a large number of experiments were performed with actual airborne InSAR data, and a set of data was selected to illustrate the effectiveness of the method in this paper. The collection interval of this data was about one day—that is, the temporal baseline was one day—while the spatial baseline was about two meters. Moreover, the mission images contained the types of human activities that we were interested in.

### 4.1. Study Area

**Figure 4:**
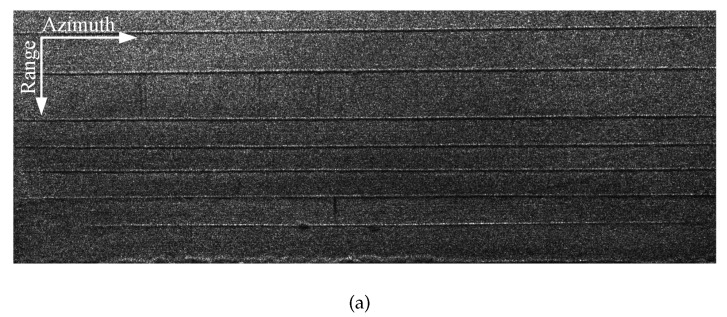

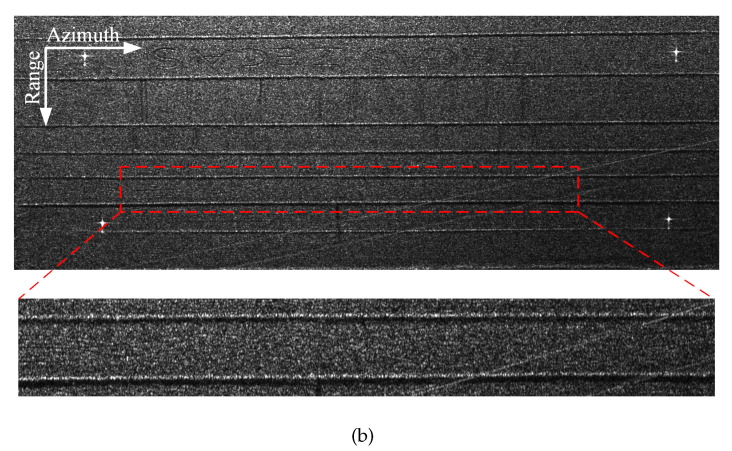
The SAR images of the experimental scene. (**a**) Reference SAR image. (**b**) Mission SAR image and enlarged image showing human activity.

The mission and reference SAR images of experimental scenes containing different types of human activities are shown in Figure 4. The average flight height at which the SAR images were obtained was about 286 m, and the image size was 2500 * 900 pixels. Since human activity involves very small changes, the changes in their amplitude information were very small. In addition, the size of human activity generally is also very small, which makes it impossible to detect human activity from the difference in amplitude information between the mission and reference images. However, phase information is another important information of SAR images that can be used for the detection of human activity. This is because the phase information is highly sensitive, especially regarding changes of the target. Generally, the change scale of the wavelength level or even the sub-wavelength level is enough to cause the phase information to be completely changed. In this paper, we utilized the phase information to detect the subtle human activities of interest.

**Figure 5:**
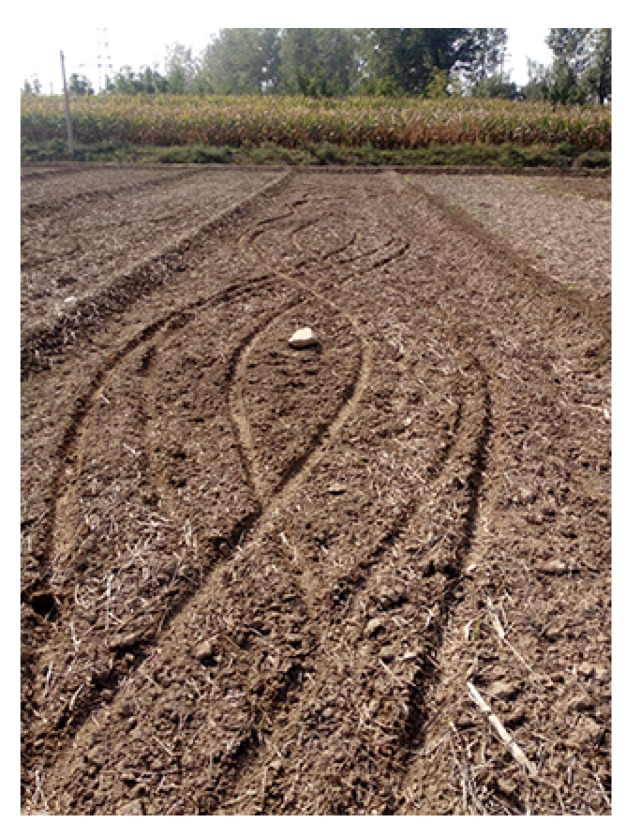
The optical image of human activity represented by vehicle tracks.

It can be seen from Figure 4b that human activity cannot be seen from the amplitude image. This means that the human activity did not result in a change in amplitude information, which was a result of the small size of vehicle tracks. The optical image of human activity represented by vehicle tracks is shown in Figure 5 as well.

### 4.2. Experimental Results for Human Activity Detection

Because the antenna beam in the azimuth direction had a certain width and the information collected in the azimuth direction was the sum of the scattering information of the target within a large range of angle, it was difficult to maintain the coherence of the target, especially in the repeat-pass interferometric observation mode. The target had different coherence characteristics in different ranges of angles. High coherence characteristics may only have been shown in a specific range of angles, with low coherence for other ranges of angles. Based on this, we introduced sub-aperture processing to the coherence improvement for human activity detection in order to obtain experimental results that could meet the requirements of human activity detection.

It was difficult for an airborne repeat-pass interferometric SAR system to maintain the same attitude and position during the two data acquisitions, which caused the coherence of the acquired data to be very low. At the same time, there were many other decorrelation factors under repeat-pass InSAR acquisition mode, such as spatial baseline decorrelation, temporal decorrelation and Doppler decorrelation, etc. It was difficult to utilize the acquired data directly for human activity detection, so it was necessary to improve the coherence between repeat-pass interferometric image pairs.

**Figure 6:**
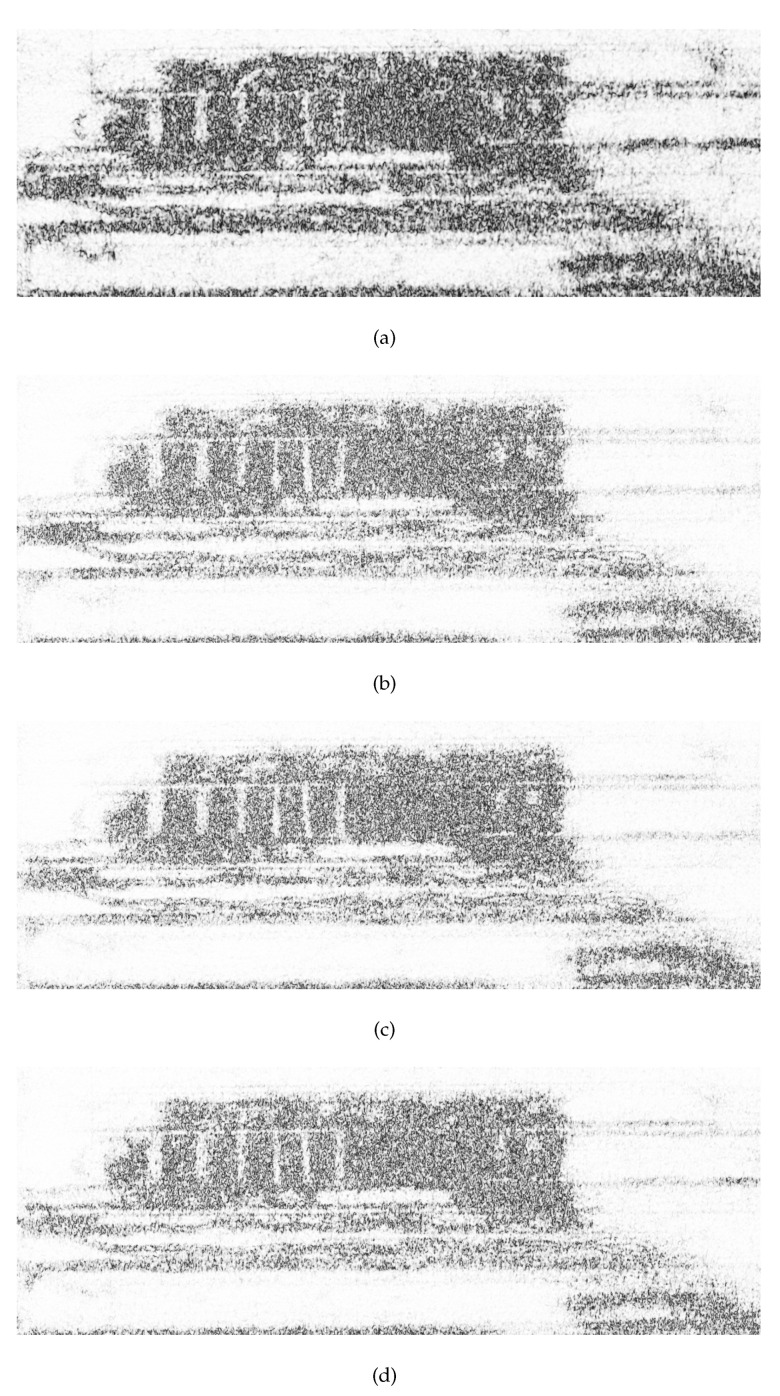
The corresponding sub-aperture coherence coefficient map of the experimental scene. (**a**) The coherence map of sub-aperture 1. (**b**) The coherence map of sub-aperture 2. (**c**) The coherence map of sub-aperture 3. (**d**) The coherence map of sub-aperture 4.

**Figure 7:**
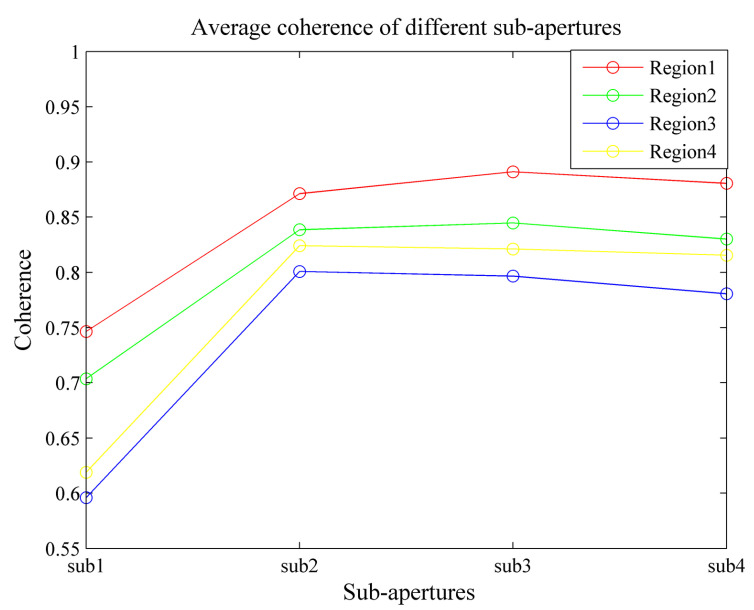
The average coherence of the target scene for different sub-apertures.

Sub-aperture processing obtains the scattering information of the target in different ranges of angle. In order to better validate the effectiveness of the sub-aperture processing in this paper, one set of repeat-pass observation data was chosen for processing. The sub-aperture InSAR processing was performed on the actual airborne experimental data in this paper. The reference and mission image were equally divided into four sub-apertures, respectively, and the results of the corresponding sub-aperture coherence coefficient map of the experimental scene are provided in Figure 6. It can be observed from Figure 6 that the coherence coefficient map of the area from different sub-apertures did not have the same distribution as a result of the disparate scattering characteristics in different sub-apertures. To validate the idea of the paper effectively, the region of interest shown in the enlarged part of Figure 4b was divided into four regions along the azimuth direction. The average coherence of each area was studied in different sub-apertures. As shown in Figure 7, the average coherence of each area varied in different sub-apertures. This was due to the SAR system obtaining distinct scattering information of the target in different ranges of angles. Thus, the coherence of the target was different between sub-apertures. The coherence difference in different sub-apertures reached 0.19 at most. The coherence of the target scene could be improved by integrating the coherence in different sub-apertures.

**Figure 8:**
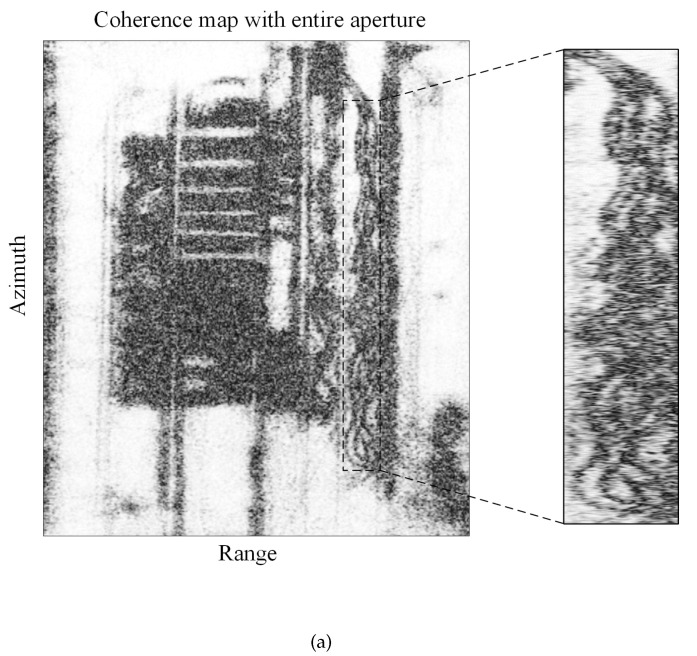

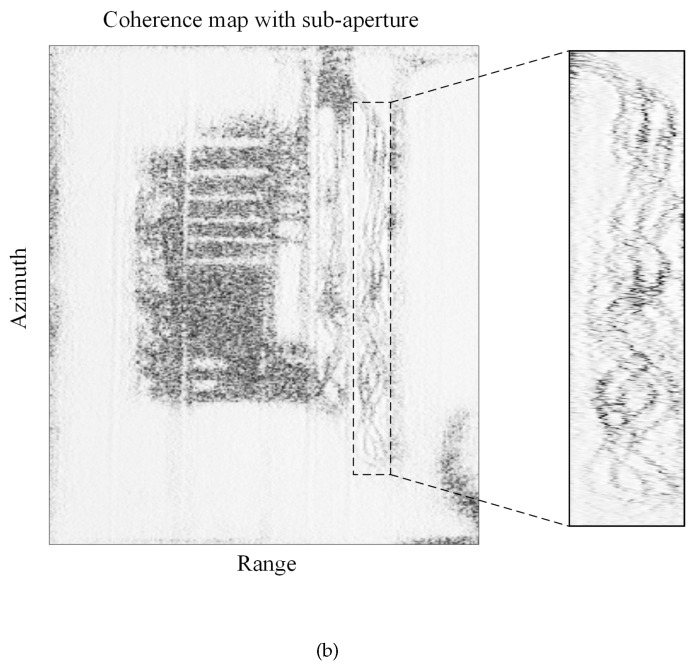
Comparison between traditional method and the proposed method. (**a**) The coherence coefficient map obtained by traditional entire aperture InSAR processing. (**b**) The final coherence coefficient map obtained by sub-aperture InSAR processing.

In order to illustrate the effectiveness of the proposed method in this paper, the results obtained by the proposed method were compared with traditional coherent processing methods. The results of two methods are shown in Figure 8. The result obtained with the traditional coherent processing method, utilizing the entire aperture between the mission and reference images, is provided in Figure 8a. It can be seen from Figure 8a that the coherence between the mission and reference images is poor. The human activities of interest are blurry in the coherence coefficient map and it is difficult to detect human activities. For comparison, the result obtained by the proposed method is shown in Figure 8b. Human activities in Figure 8b are more distinct than in Figure 8a.

The result of sub-aperture InSAR processing is shown in Figure 8b. It is obvious that there are many types of human activity, especially tricycle tracks, that are of interest. However, without sub-aperture processing, the coherence of the target scene is poor, as shown in Figure 8a. Therefore, there is no doubt that the coherence of the target scene is improved with the sub-aperture processing. Moreover, the enlarged part in Figure 8a,b on the right is the region of interest. Black areas are interesting human activities caused by the movement of a tricycle. The optical image that was taken in the target scene is shown in Figure 5. Comparing the results of the two methods, it can be seen that the result obtained by the proposed method in Figure 8b can indicate human activities of interest more clearly; thus, the improved results are more suitable to detect human activities.

**Figure 9:**
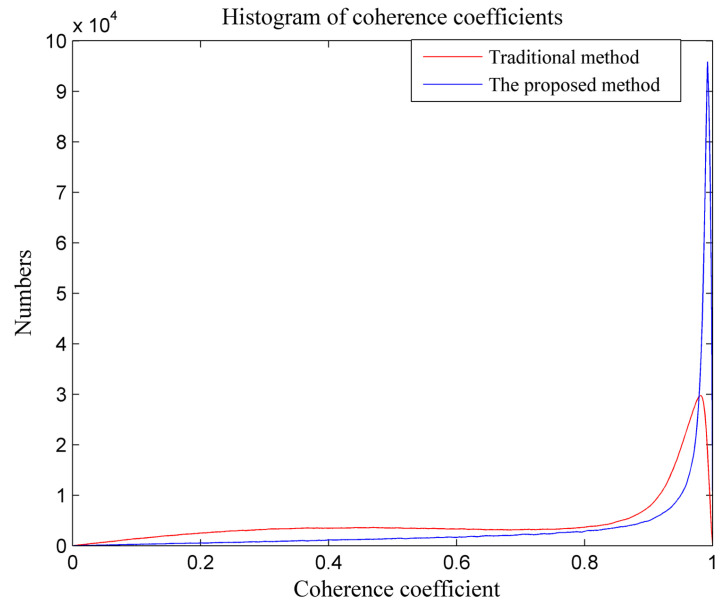
Histogram of coherence coefficients under the traditional method and the proposed method.

In order to further illustrate the performance of the proposed method, the histogram of coherence coefficients under the traditional method and the proposed method is provided in Figure 9. It can be seen from Figure 9 that the coherence was improved by the proposed method. The coherence value at the peak position of the proposed method was slightly larger than that with the traditional method. To demonstrate the effectiveness more clearly, the statistical result of the coherence coefficient is provided in Table 2. Comparing the interferometric processing result with the traditional method, the coherence with the proposed method was slightly improved. This fully confirms that the sub-aperture InSAR processing made a difference in increasing the coherence between repeat-pass image pairs. In summary, the coherence of an experimental scene can be improved with sub-aperture processing, meaning that it is beneficial for the detection of human activity.

## 5. Discussion

The FMCW SAR system has the advantages of a large bandwidth, small volume, light weight, etc. Moreover, following the rapid development of UAVs and light and small airborne platforms, the FMCW SAR system is able to carry out more applications than traditional SAR systems. This paper utilized the FMCW SAR system to perform human activity detection, and its potential was further explored regarding the use of phase information to detect human activity. In addition, due to the large number of sources of decorrelation in the airborne repeat-pass interferometric SAR system, we adopted a coherence improvement method method based on sub-aperture InSAR processing, successfully detecting human activity. Finally, the method was validated by actual airborne InSAR data. The methods and results in this paper are helpful for improving the FMCW SAR system and its applications.

Some future challenges of our work are presented below.

- Since sub-aperture images contain various scattering information of the target in different ranges of angles, the change information is also different. In order to detect human activities more completely, we will conduct studies in “change detection” between distant sub-apertures in the future.
- Since millimeter wave InSAR has unique advantage in terms of detecting subtle human activities, we will conduct further research into residual motion errors for millimeter wave InSAR. In order to compensate for complex residual motion errors, a more suitable method will be studied in the future.
- In order to reduce the influence of disturbance in the final detection results, we plan to increase the observation information of the target, and we will perform further research into the processing method of multi-pass InSAR images.

## Figures and Tables

**Table 1:** Main parameters of Ka-band frequency modulated continuous wave (FMCW) InSAR system.

Parameters	Value
Carrier frequency	34.6 GHz
Wavelength	8.7 mm
Bandwidth	2.0 GHz
Pulse repetition frequency	2000 Hz
Duration	0.5 ms
Center look angle	45${}^{\circ }$
Azimuth bandwidth	763 Hz
Platform velocity	31.6 m/s
Average flight height	286 m
Minimum slant range	434.4 m
Azimuth beam width	4${}^{\circ }$
Range beam width	16${}^{\circ }$
Slant range pixel spacing	0.0374 m
Azimuth pixel spacing	0.0242 m

**Table 2:** The statistical results of coherence coefficients.

	γ>0.5	γ>0.7	γ>0.9	Average
Traditional method	72.09%	57.34%	38.54%	0.696
Proposed method	80.58%	70.93%	53.72%	0.802

## Data Availability

Data sharing not applicable.
